# Relating the Chondrocyte Gene Network to Growth Plate Morphology: From Genes to Phenotype

**DOI:** 10.1371/journal.pone.0034729

**Published:** 2012-04-30

**Authors:** Johan Kerkhofs, Scott J. Roberts, Frank P. Luyten, Hans Van Oosterwyck, Liesbet Geris

**Affiliations:** 1 Biomechanics Research Unit, University of Liège, Liège, Belgium; 2 Biomechanics section, K.U. Leuven, Leuven, Belgium; 3 Prometheus, The Leuven R&D division of skeletal tissue engineering, K.U. Leuven, Leuven, Belgium; 4 Rheumatology Department, K.U. Leuven, Leuven, Belgium; The University of Hong Kong, Hong Kong

## Abstract

During endochondral ossification, chondrocyte growth and differentiation is controlled by many local signalling pathways. Due to crosstalks and feedback mechanisms, these interwoven pathways display a network like structure. In this study, a large-scale literature based logical model of the growth plate network was developed. The network is able to capture the different states (resting, proliferating and hypertrophic) that chondrocytes go through as they progress within the growth plate. In a first corroboration step, the effect of mutations in various signalling pathways of the growth plate network was investigated.

## Introduction

Recent advances have elucidated the regulatory mechanisms of endochondral ossification, where bone is formed through a transient cartilage anlage. During this process cells exhibit distinct and easily observable differentiation stages, as is readily evidenced by the morphology of the growth plate [1].

The growth plate is a developmental centre that integrates many signalling pathways in order to regulate the patterning and growth of long bones. As the cells progress throughout the growth plate, going from the long bone’s epiphysis towards the diaphysis, their shape and function change drastically [2]. At the epiphysis, a pool of small round chondrocytes makes up the resting zone. These cells differentiate into more rapidly proliferating flat chondrocytes, forming proliferative columns. The resting and proliferating chondrocytes secrete structural proteins, such as collagen type II, that form a hyaline cartilage matrix. Towards the diaphysis, chondrocytes differentiate further into prehypertrophic and thereafter hypertrophic chondrocytes [3]. Hypertrophic chondrocytes remodel the cartilage matrix into a calcifying matrix comprising primarily collagen type X. At terminal differentiation, the hypertrophic cells will induce invasion and resorption of the mineralized cartilage matrix as well as the start of vascularisation by secreting a specific set of proteins like MMP13 and VEGF [4].

The growth plate chondrocytes must respond to positional cues, local agents and hormonal signals to coordinate the formation of unique skeletal elements [5]. Important local signalling pathways regulating the endochondral development of bones are the parathyroid hormone related peptide (PTHrP), Indian hedgehog (Ihh) [6], bone morphogenic proteins (BMPs) [7], transforming growth factors β (TGFβs) [8], Wnts [9] and Fibroblast growth factors (FGFs) [10]. These pathways exert their influence on the growth plate, at least in part, by regulation of the key transcription factors Sox9 [11] and Runx2 [12]. The former is crucial for chondrogenesis, whereas the latter is a central regulator in chondrocyte hypertrophy.

Ihh and PTHrP form a feedback loop that regulates the length of the proliferative column. Prehypertrophic chondrocytes exiting the proliferative pool express Ihh. Through unknown means this Ihh signals to periarticular chondrocytes to produce PTHrP. This PTHrP will suppress chondrocyte hypertrophy by binding its receptor PPR (Parathyroid hormone/PTHrP Receptor) and prevent Ihh expression until the proliferative chondrocytes leave the PTHrP signalling range [13].

Several methods to model gene networks are widely used, ranging from more mechanistic models to entirely empirical methods. The former include detailed thermodynamic approaches capable of dealing with limited molecule numbers or the mean-field approximation of ordinary differential equations based on the law of mass action or other principles (reviewed in [14]). The latter include methods such as network inference by correlation, regression and Bayesian techniques [15]. Given the complexity and high interdependency of the signalling pathways in endochondral ossification, we set out to take a logical (multi-value Boolean) approach to model the developmental process. A logical model is highly practical to structure and research this intricate system of control mechanisms. This approach has the added advantage that no exact knowledge of the concentrations and reaction rates of the factors used by the relevant signalling cascades is needed, since these data are not readily available in the literature. For modelling the growth plate, the logical formalism hence represents a good compromise between the highly detailed dynamics of mechanistic models and the black box approach of data-driven phenomenological models [16]. As this work focuses on the growth plate as an autoregulatory semiautonomous module, the model includes only autocrine and paracrine signalling pathways. The purpose of this model is to examine the individual and combined influence of relevant growth factors and their subsequent signalling cascades on chondrocyte differentiation in the growth plate.

## Materials and Methods

**Figure 1:**
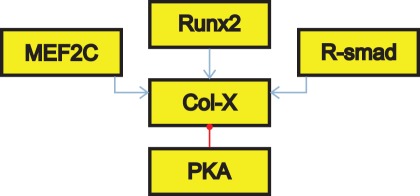
Regulation of Col-X. Col-X is regulated by several factors, each symbolized by a node (yellow square). Arcs represent interactions of two kinds: an activating interaction is represented by a blue arrow, whereas an inhibitory one is given by a red line with a dot.

The network consists of a directed graph where biological factors and their interactions are represented by nodes and arcs respectively [17]. Each arc is characterized by a sign, as seen by a different color and shape in Figure 1. The sign of these arcs determines an activating (positive) or an inhibitory (negative) effect. Every arc is furthermore associated with a certain activity range. This range indicates at which levels of the activating node the interaction is active. To simulate the dynamics of the model, every node is associated with a logical function that will set its value based upon the active interactions. This function associates a value (called parameter in Chaouiya et al. [18]) with every possible set of active interactions, hence determining the next value of the node. A logical function is fully specified by a truth table, an example of which can be found in Table 1. The left column specifies which inputs are active, and the right column attributes a value accordingly. Table 1 contains the truth table of the logical function specifying regulation of collagen type X (Col-X), as graphically presented in Figure 1. R-smad, Runx2 and MEF2C have been shown to stimulate Col-X transcription [19]. PKA on the other hand inhibits Col-X transcription [20]. The Runx2 and Col-X nodes can have a level up to two, while the other nodes have a maximum of one. This use of multi-value logic allows the model to more accurately capture gradients in the growth plate. In addition, multi-value logic allows the effects of interactions that only partly disable (or activate) a node to be included.

**Table 1:** Logical function describing the value of node Col-X.

Active interactions	Output of logical function
Runx2(2), MEF2C(1)	1
Runx2(2), R-smad(1)	1
Runx2(2), R-smad(1), PKA(1)	0
Runx2(1), MEF2C(1), R-smad(1)	1
Runx2(2), MEF2C(1), R-smad(1)	2
Runx2(2), MEF2C(1), R-smad(1), PKA(1)	1

The Runx2(2) interaction is active when Runx2 has a value of 2, while the Runx2(1) interaction indicates a Runx2 value of 1. The interaction combinations not shown in the table have a value of zero.

Wherever possible the logical function was deduced from literature. However, deduction of appropriate values is problematic due to the lack of sufficient literature information, especially in cases where multiple interactions are simultaneously active. In these circumstances the strength of the different interactions is taken to be identical, since no data exists on the relative strengths of gene interactions. Hence the state of a node is determined somewhat semiquantitatively, and will be active when there are more stimulatory interactions and inactive in the case of a majority of inhibitory interactions. This is a weighted sum approach, which has been applied in systems of ODEs [21], feature extraction [22] and Boolean networks [23]. In the Boolean formalism, functions of this type have been termed ‘additive functions’ or ‘majority functions’ and have been shown to be biologically relevant [22]–[24]. In the example of Col-X regulation only when the stimulatory interactions are all active and the inhibitory interaction is not, the maximum value is attributed to the Col-X node. If one of the stimulatory interactions is absent or the inhibitory interaction is active the node is given a value of one. In all other situations the node is considered inactive.

Nonetheless information on the nature of the interaction was frequently available, allowing the values of the logical function to be further refined. For example, one gene (or rather its protein product) might activate another through phosphorylation, while others might increase that genes’ activity through transcriptional mechanisms. Clearly posttranslational modifications like phosphorylation and ubiquitination will only matter if the gene is transcribed in the first place. In this logic the activity of a gene will be penalized when it is not activated through phosphorylation or when it is not effectively transcribed (e.g. a majority of inhibitory transcription factors is present).

**Figure 2:**
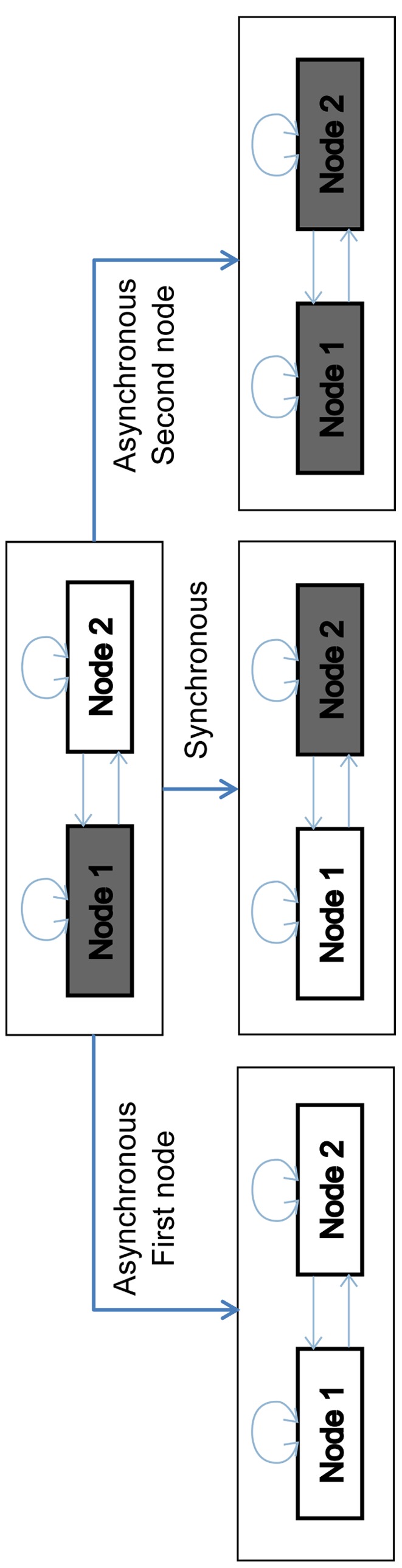
Synchronous versus asynchronous updating strategy. This example clarifies how the use of different updating strategies can change the dynamics of the system. Grey and white color indicate the node has a value of 1 and 0, respectively. Each node is regulated by its own value and that of the other. Node 1 is governed by an AND gate, i.e. it is 1 only when both inputs are 1. Node 2 is regulated by an OR gate, it is 1 when either of its inputs is 1. Under asynchronous updating, the system will reach a different stable state depending on which node is updated first. When both nodes are updated synchronously yet another stable state, unattainable by asynchronous updating, is reached.

The state of the network is determined by an n-tuple containing the values of all nodes. The dynamics of the model is simulated with a dynamical graph. In this graph, nodes represent the state and the arcs represent spontaneous transitions between the states. The dynamics of the graph can be either synchronous or asynchronous. In synchronous dynamics all nodes are updated at the same time. This means that every interaction is assumed to have an equal duration, which of course is not realistic in biological systems. In asynchronous dynamics multiple updating orders do not occur simultaneously and a criterion is needed to determine priority. Hence, each interaction is associated with a certain time delay. When no information on the time delay is provided, as within this study, all possible transitions are generated. As a consequence, each state has a number of successors equal to the number of nodes to be updated at this state. An example is given in figure 2. Ultimately the gene regulatory network will reach a steady state which has no successor distinct from itself. These states are called singleton attractors. Alternatively, the network can oscillate in a dynamic cycle termed cyclic attractor, typical for phenomena like circadian rhythms [25].

Synchronous updating can give rise to modelling artefacts by creating spurious cyclic attractors [26], [27]. By contrast, singleton attractors are stable regardless of the (discrete) updating mechanism. Given the lack of kinetic information, we use synchronous updating assumptions. However, as seen in figure 2, an initial state may flow to a different stable state depending on the updating mechanism. For this reason, we choose to also investigate the entire transition graph under asynchronous updating. Furthermore, we only consider singleton attractors to avoid the previously mentioned modelling artefacts.

The described approach permits the use of multi-value logic. This allows modelling several distinct active states so that the levels can represent different biological effects. All growth factors (with the exception of TGFβ), their receptors and crucial transcription factors (Runx2, Sox9, β-catenin, Nkx3.2, Smadcomplex) as well as the output genes Col-X and Col-II were implemented with multi-value logic. Further details on this logical formalism can be found in Chaouiya et al. [18]. The formalism is implemented in GINsim 2.3, a java application designed to model and simulate genetic regulatory networks [28] and the model files are provided as Material S2.

The knockout of a certain gene is represented by deactivation or removal of the protein, which is achieved by setting the value of its node to zero. Constitutive expression of a gene can be represented by setting the value of the associated node to the highest activation level (on-state).

Spatial migration through the growth plate cannot be captured by logical networks but was modelled in this study by altering the expression level of external ligands (PTHrP, TGFβ & some FGFs) that are expressed locally in the perichondrium surrounding the growth plate. The presence or absence of these ligands hence serves as an input. These values are not determined by the network but influence its outcome.

The logical network presented here is based on an extensive literature study. The data are mostly taken from studies of the fetal growth plate in mice and chick models. As the network is cell type-specific only interactions shown to occur in growth plate chondrocytes were included, unless no such information was available, in which case data were taken from related cell types such as osteoblasts. The network incorporates the effects of the major paracrine signalling pathways on Sox9/Runx2 activity. These transcription factors are crucial for early chondrogenesis and subsequent hypertrophy. Hence we hope to capture the effects of certain growth factors on these processes by modelling the factor’s influence on their most crucial mediators. The model consists of 35 nodes and 111 interactions. For details on the included signalling pathways and their crosstalks the reader is referred to the given references and references therein. A list of interactions and the references from which they were derived is provided (Material S1).

To assure robustness of our results we also created an ODE version of our model, an approach similar to that of Mendoza et al. [29], that allowed us to numerically check whether the logical stable states where stable if the logical assumptions were relaxed. Practically, we replaced the logical step functions with progressively gentler sigmoids (see Material S3 for model equations and further details). This analysis confirmed the robustness of our results for the stable states of the wild type growth plate (see Material S3). Though analytical approaches exist [30], given the high number of nodes and thresholds in our model, their application would be cumbersome.

## Results

### Prediction of Growth Plate Dynamics

**Figure 3:**
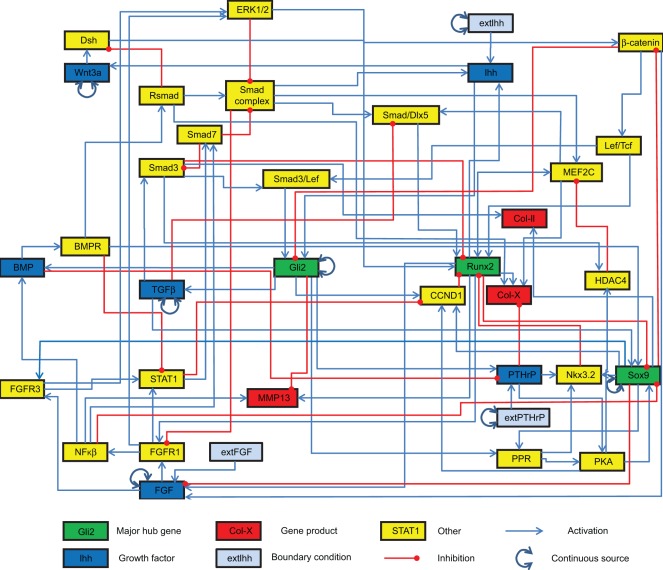
The growth plate chondrocyte gene regulatory network. Every node in the gene network is represented as a square, the interactions are represented by arrows between squares. The network as shown here was coarse grained, i.e. nodes not influenced by multiple reactions were omitted. Therefore linear signalling cascades are not represented here.

**Figure 4:**
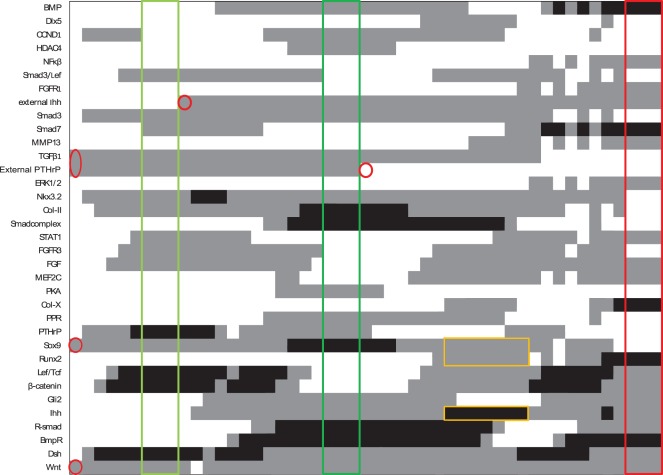
Representation of growth plate dynamics. The expression values of each node as the network progresses from one stable state (framed columns) to the next by changing the inputs (indicated by a red circle). White, grey and black respectively indicate values of 0, 1 and 2. The resting (green), proliferating (dark green) and hypertrophic (red) stable states are indicated. As noted, the prehypertrophic state (yellow rectangles) is not stable but is a transient state between the proliferating and hypertrophic states. It is characterised by simultaneous expression of Runx2 and Sox9 and increased Ihh expression.

**Figure 5:**
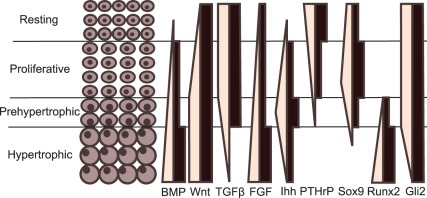
Predicted versus observed expression patterns in the growth plate. The literature derived (light) and predicted (black) expression patterns in 4 growth plate zones. BMP ligands become more abundant as chondrocytes mature [66] as is also the case in the modelled gene network. The expression pattern of Wnt4a [46] is shown here to represent the canonical Wnts, this pattern also closely resembles that of active β-catenin which is the canonical Wnt signal transducer [67]. TGFβ is expressed throughout the growth plate, except in the hypertrophic zone [8]. FGFs are excreted in increasing amounts in more mature cells under influence of Runx2 [68], [69] PTHrP and Ihh are expressed by resting zone and prehypertrophic chondrocytes respectively [5]. Runx2 expression increases as chondrocytes hypertrophy [70], while Sox9 activity reaches its peak in proliferative chondrocytes [71]–[73]. Gli2 is also continuously expressed in the growth plate, but its expression tapers off in hypertrophic cartilage [74].

The growth plate network is given in Fig. 3. It describes the influence of important paracrine or autocrine growth factors (blue nodes) on cell differentiation as represented by the activation of crucial transcription factors (green nodes). The result of a synchronous simulation with conditions that match those seen in the growth plate *in vivo* is depicted in Figure 4. The stable states generated by the network results are shown in Table 2. The spatial configuration of key transcription and growth factors in the growth plate is shown in Figure 5.

**Table 2:** Stable states of the wild type network.

		Growth factors					Transcription factors		
Zone	BMP	Wnt	TGFβ	FGF	Ihh	PTHrP	Sox9	Runx2	Gli2
**Resting zone**	0	1	1	1	0	2(+ext)	1	0	0
**Proliferative zone**	1	1	1	1	1(ext)	1(ext)	2	0	1
***Prehypertrophic zone***	1	1	1	1	2(+ext)	0	1	1	0
**Hypertrophic zone**	2	1	0	1 (+ext)	1(+ext)	0	0	2	1

The values of nodes representing growth factors and transcription factors are tabled for every zone in the growth plate. As the Ihh/PTHrP feedback loop involves diffusion of ligands from the prehypertrophic zone into the resting zone and vice versa, the nodes in the network indicate the presence of diffused Ihh and PTHrP respectively. Furthermore, FGF18 diffuses from the perichondrium to bind FGFR3 in proliferating chondrocytes [9]. The presence of paracrine signalling is indicated in the table (ext or +ext if auto- and paracrine signalling are mixed).

In the network, the prehypertrophic zone does not normally form a stable state. In early hypertrophy, Wnt and FGF have a synergistic effect and upregulate each other’s ligands. This leads to further upregulation of Runx2 and fastens hypertrophy. When BMP signalling is sufficiently hampered by FGF activity, the expression of Ihh, typical for the prehypertrophic zone, will be lowered. Hence every chondrocyte undergoing hypertrophy will highly express Ihh for a limited amount of time, between the onset of Runx2 expression and the decrease in canonical BMP signalling. However, the discrete time step of the logical model does not directly correspond a real time step. For this reason, we cannot estimate how long this expression would persist nor how dependent this state is on the actual speed of interactions. In order to correctly model expression in the resting zone, the induction of PTHrP by Ihh was assumed to be indirect (see discussion for more detail).

### Simulation of Mutant Cases

The network can be validated by and used to investigate certain knockouts or activating mutations of key players of growth plate dynamics. We have carried out simulations of mutants in each of the 5 major pathways included: the Ihh/PTHrP feedback loop, FGF, BMP, Wnt and TGFβ signalling. In the case of the Ihh/PTHrP feedback loop, the outcome of a disabled and constitutively active PTHrP pathway is examined. In the PTHrP null network, the situation within the resting zone is the same as in wild type mice except for a lack of PTHrP production. The relevant stable states are shown in Table 3. Since PPR is not expressed in this zone the network behaviour is unchanged. When in the proliferating zone external Ihh induces BMP expression, the network will enter a hypertrophic state as a consequence of absent PTHrP signalling. The network hence predicts that chondrocytes will undergo hypertrophy faster, as the stable state characterizing a proliferative chondrocyte is skipped. By calculating the transition graph it becomes clear that under asynchronous updating these stimuli can induce either a Sox9 positive state or the aforementioned state of hypertrophy. Which state will be reached *in vivo* is determined by the unknown pace of the interactions represented by the network arcs. More specifically, in the real biological network every interaction has a certain rate, which can be related to how fast a reaction should be updated. The state that is achieved by first updating nodes that are under control of faster interactions, such as phosphorylation, is more likely to be biologically relevant.

**Table 3:** Stable states of the PTHrP mutant networks.

		Growth factors					Transcription factors		
PTHrP-	BMP	Wnt	TGFβ	FGF	Ihh	PTHrP	Sox9	Runx2	Gli2
**Resting zone**	0	1(ext)	1(ext)	1	0	0	1	0	0
**Hypertrophy** **(synchronous)**	2	1	1	1	1	0	0	2	1
**Sox9 positive (asynchronous)**	1	1	1	1	1	0	1	0	1
**PTHrP+ (PPR+)**	**BMP**	**Wnt**	**TGFβ**	**FGF**	**Ihh**	**PTHrP**	**Sox9**	**Runx2**	**Gli2**
**Resting zone**	0	1	1	0	0	2	1	0	0
**Proliferative**	1	1	1	0	1(ext)	2	2	0	1
	1	1	0	2	1(ext)	2	2	0	1
**Hypertrophy**	1	1	0	1	1	1	0	1	1

The values of nodes representing growth factors and transcription factors are tabled for the relevant stable states in the mutant growth plate. The presence of paracrine signalling is indicated in the table (ext). For the PTHrP null network, the resting zone situation is unchanged, save for PTHrP secretion. However, a stable proliferative state is nonexistent and a state of hypertrophy is reached instead. By calculating the state transition graph a Sox9 positive state can be detected. In a network with constitutively active PPR (PTHrP+/PPR+), the resting zone is identical to the wild type situation. The proliferative zone is also unaffected. However, no hypertrophy is reached as the PTHrP pathway remains active and hence blocks chondrocyte differentiation. Hypertrophy might be reached if PTHrP signalling is diminished.

In the model with a constitutively activated PPR expression (identical to ectopic expression of PTHrP) the situation in the resting zone is unchanged as PPR is not expressed here (Table 3). The proliferative stable state is unaffected as well, however this is under the assumption that Ihh is present. With constitutively activated PPR the chondrocytes cannot leave the proliferative pool, even when TGFβ signalling, also inhibiting hypertrophy, is assumed to be absent. However, an alternative stable state with moderate Runx2 activity is possible if PTHrP expression is not maximal. This can be observed experimentally in mice expressing PTHrP under the control of a Col-II promoter (Table 3). In these mice PTHrP expression mirrors Col-II which is expressed to a lesser extent in some areas, notably on the fringes of the long bone [31].

For the FGF pathway, we simulated an overactivation of the fibroblast growth factor receptor 3 (FGFR3) (Table 4). Achondroplasia can be simulated by forcing the FGFR3 node to be maximally activated. The FGFR3^ACH^ mutant does not change chondrocyte behaviour in the resting zone, the increased FGF signalling has little effect here as there is no BMP and wild type chondrocytes do not proliferate in this zone. The more activated ERK pathway might stimulate Runx2 activity, but the activation of the ERK node is insufficient to change Runx2 expression. In the proliferating zone BMP ligands allow for more active Sox9. However, due to dominant FGF signalling Sox9 is less activated in the FGFR3^ACH^ proliferating chondrocytes. Furthermore, the CCND1 gene, which is associated with an increased proliferation rate [32], is not expressed (not shown). When perichondrial TGFβ signalling diminishes, the chondrocytes will undergo hypertrophy. The way this hypertrophy is achieved is different than in the case of the normal network, where MEF2C, a transcription factor regulating hypertrophy [33], will be expressed before Runx2. In the mutant network this sequence is reversed. Additionally, due to the absence of the Smad complex, a lower amount of Ihh secretion is predicted. The stable states of the FGFR3 mutant are shown in Table 4.

**Table 4:** Stable states of the FGFR3 mutant network.

	Growth factors						Transcription factors		
FGFR3+	BMP	Wnt	TGFβ	FGFR3	Ihh	PTHrP	Sox9	Runx2	Gli2
**resting zone**	0	1	1	2	0	2(+ext)	1	0	0
**proliferative**	1	1	1	2	1(ext)	1(ext)	*1*	0	1
**hypertrophy**	2	1	0	2	1	0	0	2	1
**Smad KO**	**RSmad**	**Wnt**	**TGFβ**	**FGF**	**Ihh**	**PTHrP**	**Sox9**	**Runx2**	**Gli2**
**resting zone**	0	1	1	1	0	2	1	0	0
**apoptosis**	0	1	0	2	1(ext)	0	0	0	0
**hypertrophy**	0	1	0	2	1	0	0	2	0

The values of nodes representing growth factors and transcription factors are tabled for the relevant stable states in the R-Smad and FGFR3 mutant growth plate. The presence of paracrine signalling is indicated in the table (ext). The situation in the resting zone is virtually unchanged. However, the activity of Sox9 in the proliferation zone is hampered. The stable state representing hypertrophy is also unaffected. For the Smad mutant the proliferative zone is more severely impacted and no expression of Sox9 is predicted. The hypertrophic state can be reached under certain asynchronous updating assumptions, as is apparent from the state transition graph.

The BMP pathway is tested by simulation of a Receptor-regulated Smad (R-Smad), the canonical effectors of BMP signalling [7], knockout. The resting zone is unaffected, as BMP ligands are not present there (Table 4). However, the situation of proliferating zone chondrocytes will not differ much from those of the resting zone. PPR is not activated in the mutant network, which results in a reduced proliferation rate and a lowered Sox9 expression. This state is similar to the resting zone state. If perichondrial signalling alters to promote hypertrophy, i.e. no expression of TGFβ and increased FGF expression, the cells will not enter a hypertrophic phase but they will lose Sox9 expression. By calculating the transition graph, it is observed that two possible stable states can be attained. One state is the same as the one reached by synchronous updating, the other is a state of hypertrophy with production of Ihh. The results for the Smad mutant are listed in Table 4.

## Discussion

The network constructed in our study qualitatively captures the behaviour of growth plate chondrocytes in response to the modelled signalling pathways. The network is able to capture the different stable states (resting, proliferating and hypertrophic) that the chondrocytes transcend as they progress through the growth plate [3], [4]. The prehypertrophic state was predicted to be an unstable state at the transition between proliferation and hypertrophy.

It is unknown whether Ihh regulates PTHrP expression in the periarticular chondrocytes in a direct or indirect fashion. In the model presented in this paper, the induction of perichondrial PTHrP by Ihh was assumed to be indirect since the network perspective of the growth plate presented in this paper speaks against direct control of PTHrP by Ihh. Firstly, Ihh target gene Ptch1 expression is strongest in the domain adjacent to the Ihh domain, fading away towards the articular surface [6], [34]. This shows that the concentration of Ihh in the periarticular region will be minimal, if any. Secondly, Ihh has been shown to induce BMP in chondrocytes, while BMP is not expressed in the resting zone [35], [36]. Hence the assumption of secondary signals prevents the prediction of non-physiological BMP expression in periarticular chondrocytes and consequently a lower Sox9 activity in the resting zone. The absence of BMP molecules in the resting zone can alternatively be explained by assuming that Ihh-dependent BMP production only takes place once a certain threshold concentration of Ihh is reached. This would mean that Ihh can stimulate PTHrP production at much lower concentrations then required for BMP expression. However, no such dependency has been reported.

Therefore we have assumed Ihh signals to the resting zone by a secondary factor, more specifically by ligands excreted in the proliferating zone perichondrium. Signals from perichondrial cells themselves might be responsible rather than diffused Ihh ligands since Ptch1 expression can be detected in these cells, indicating that Ihh indeed exerts an influence upon perichondrial cells [37]. Perichondrial cells excrete TGFβ and Wnt ligands which could in turn diffuse to periarticular chondrocytes to induce PTHrP ligand secretion [38], [39]. TGFβ signalling (in the perichondrium) may mediate the Ihh/PTHrP feedback loop by acting upstream of PTHrP but downstream of Ihh [39]. Decapentaplegic (Dpp, analogue of TGFβ) mediates Hh signalling in Drosophila although TGFβ is certainly not a universal target of the pathway [40] but microarray data indicates Gli can upregulate TGFβ in chondrocytes [35]. All TGFβs stimulate PTHrP expression in chondrocytes, presumably through Smad-dependent mechanisms. These data indicate that TGFβ is a plausible messenger downstream of Ihh. Of note, only less differentiated cells can express PTHrP [41]. However, the ensemble of factors responsible for this difference requires clarification.

PTHrP expression might additionally be regulated by BMP since in osteoblasts BMPs can inhibit PTHrP expression. Accordingly BMPs may play a similar role in proliferating chondrocytes [42]. Interestingly, in Ptch1^-/-^ mouse where Hh signalling activity is always at maximum the PTHrP expression pattern does not change, indicating some other factor, independent of Ihh, must be responsible for this localization [43]. This discussion shows that while the Ihh/PTHrP feedback loop has been extensively studied, the exact mechanisms through which Ihh controls PTHrP require further investigation.

Although we opted not to include noncanonical Wnt signalling, a clear cut distinction between both types is hard to make as a marked delineation between canonical and noncanonical Wnts might be idiosyncratic and could easily be blurred by varying circumstances such as tissue type, differentiation state of cells, the availability of receptors on the cell surface and the presence of other factors in the cell lumen [44]. The focus of research efforts in chondrocytes thus far has been more on β-catenin/TCF signalling, providing a relative abundance of information on canonical signalling when compared to knowledge on the mechanisms referred to as noncanonical signals. Be that as it may, some workers have concluded that noncanonical Wnt5a and Wnt5b cooperate to antagonize hypertrophic differentiation and hence β-catenin/TCF signalling, although which exact signalling pathways are employed remains to be elucidated [9], [45]. Hence an important caveat on the results presented here is that the incorporation of Wnt signalling in the presented network is incomplete and might lead to faulty conclusions.

In a first corroboration step the *in vivo* expression patterns of genes featuring in the network are compared to the activity of their logical counterparts. Fig. 5 evaluates the expression patterns observed in the growth plate and the modelling results. The predictions and observations match well, taking into account the discretization innate to the logical framework. Some mismatches can be found, however, such as the presence of Wnt in the resting zone. This is due to the assumption that Wnt is downstream of Ihh. The expression pattern can be rectified by presuming TGFβ to be the only Ihh messenger molecule, notwithstanding that the molecular mechanisms utilised to transcribe perichondrial PTHrP remain elusive. There is also evidence that Wnt ligands and consecutive signals are regulated by insulin-like growth factor 1(IGF-1), which was not included in the model [46].

Another discrepancy is the appearance of Gli2 in hypertrophic chondrocytes, an error that is corrected by allowing FGF signalling to inhibit Hh signalling, as was described previously [47], [48], or by letting Ptch1 expression decrease as a result of Sox9 absence [49], [50]. However, since the detailed mechanisms of these interactions remain obscure they were not included in the current model.

The mutant results can be compared to murine models as a further step towards model corroboration. Mice with a homozygous deletion of PTHrP show no abnormalities in early development, but show defects later during endochondral bone development and die shortly after birth [51]. In these PTHrP null mice the transition of chondrocytes from the proliferative to the hypertrophic phase is accelerated, resulting in advanced and premature ossification [52]. Additionally, the mice appear to be smaller at birth in comparison to their wild type relatives. This dwarfism is likely due to proliferating chondrocytes that divide less before undergoing hypertrophy. A similar phenotype is found in mice lacking PPR. Hence these mouse models corroborate the results reached by the growth plate network for the case of PTHrP deactivation. However, it remains to be seen whether chondrocytes are capable of reaching a state similar to the Sox9 positive state predicted by asynchronous updating. An activating mutation in the PPR gene has been found to cause Jansen’s metaphyseal chondrodysplasia, a disease characterized by dwarfism of the limbs and hypercalcemia [53]. A mouse model where PTHrP is overexpressed in cartilage by a Col-II promoter reveals a similar phenotype [31]. The mice exhibit a short-limbed dwarfism and are born with a cartilaginous skeleton, indicating that hypertrophy did not occur, in accordance with the model results. After about seven weeks the skeleton will have mineralized starting at the periphery of the bones, inverting the process of endochondral ossification where the bone is mineralized inside out [31]. This means that at the periphery of the bone PTHrP expression might be lower, and hypertrophy could occur here. The chondrocytes would then reach the state of hypertrophy predicted by the model in the case of lower PTHrP expression. The model was able to successfully predict the changes in the growth plate structure for the mutated PTHrP signalling.

FGFR3 is the most common aetiological factor of human dwarfism or achondroplasia (ACH). A mutation in the gene causes it to become more active. The effect of this mutation is relatively minor, since more powerful activating mutations have a lethal phenotype, such as in thanatophoric dysplasia [54]. FGFR3^ACH^ mice have been created by using regulatory elements from the collagen-II gene to transcribe an activated form of FGFR3 in the cartilage growth plate. Hence the situation in these mice is similar to the conditions simulated to corroborate the behaviour of the FGF pathway. These transgenic mice exhibit appendicular skeletal hypoplasia alongside other bone defects. Their skeletal underdevelopment seems to originate from a reduced chondrocyte proliferation combined with a slower differentiation [48]. This is also shown in the model where CCND1, an important gene in the cell cycle, is no longer expressed. The model also predicts the hypertrophic differentiation of the chondrocytes is hampered given the absence of MEF2C and Dlx5, both hypertrophy-promoting transcription factors.

In the case of a conditional knock out (CKO) of the R-Smads (1 and 5) in chondrocytes growth plate morphology is dramatically disrupted. R-Smad CKO mice exhibited an increased area with occurrence of apoptosis but a total absence of a hypertrophic zone. Furthermore, these mutants show a decreased expression of Col-II and decreased levels of Sox9 in the mutant cartilage, indicating that the chondrocytes might not be fully differentiated [55]. Accordingly, the model predicts absence of Sox9 expression at this stage. This may be linked with the increased apoptosis, as Sox9 is required for chondrocyte survival [56]. In an asynchronous updating regime the network can reach a hypertrophic state. However, the R-Smad CKO growth plate shows no apparent hypertrophic zone. Nonetheless, a low level Runx2 expression was detected by RT-PCR demonstrating a patched expression of the transcription factor in the disorganised mutant growth plate [55]. Hence a limited number of cells might reach this stable state. The other major pathways were tested in a similar way providing further corroboration (see Material S4). Together these mutant cases suggest the major pathways and their function in the growth plate can indeed be simulated adequately by the presented logical model.

It should be noted that many signalling pathways have been simplified to a generic signalling molecule representing many ligand/receptor combinations. For example, the network does not differentiate between BMP2, -4, -6 or -7, which are differentially expressed in the growth plate [5]. Neither was a distinction between BMPR1A and -1B included. The reason for this is twofold. Firstly, very few data are available on the differences between these BMP ligands, let al.one on their mutual interactions and combinations with different receptor types. Hence inclusion of these data paints an incomplete picture of BMP ligand control resulting in very erratic expression patterns, whereas their combination in a generic signal results in robust behaviour. Secondly, the dissimilarities between these interactions are often quantitative in nature, which is hard to reconcile with a logical model, which is inherently qualitative. For the purposes of qualitative predicting cell behaviour, there was consequently no impetus to include the individual ligands.

This model has several limitations. For instance, from the behaviour of the chondrocyte network it is apparent that the presence of the perichondrium is necessary to form an autoregulatory unit. Therefore a more comprehensive model could be constructed which includes differentiation of mesenchymal cells to form the surrounding perichondrium (an osteogenic lineage). A further limitation of the model is the inability to simulate the terminal differentiation phase, which would require inclusion of survival-related pathways responsible for apoptosis. A crucial consideration is that the growth plate network combines information from multiple species. While the majority of interactions were confirmed in mouse models, 18% was not. We assumed these interactions to be evolutionary conserved across vertebrates, though this remains unconfirmed. More information on the models from which interactions were derived can be found in Material S1. Moreover, the results of the model are dependent on the weighted sum approach. Using another principle such as dominant inhibition would significantly alter the results. We selected the weighted sum approach because it seemed to comply best with the kind of data available for interactions and their effect on gene expression.

Additionally, the many simplifications of logical networks have some drawbacks. Sometimes an all-or-nothing response is insufficient to describe wide range of continuous concentrations a certain protein may have. This is increasingly problematic in hub genes, where many pathways converge with often contradictory effects. This problem is partially mediated by the use of multi-value logic, as was done in this study. Furthermore, a logical network does not have any spatial or realistic temporal resolution. The dynamics of the networks are hence only roughly approximated. More specifically, caution is warranted concerning the appearance of a transitory prehypertrophic state, which may be depended on the time scale at which interactions take place. The model could be further improved by associating a time delay with every interaction, thereby enhancing time resolution [57]. However, since we lack kinetic information and a random approach is not computationally feasible due to the amount of possible time delays and combinations thereof we have limited our simulations to two cases. The first case is that of synchronous dynamics where all interactions are equally fast and in the second case we consider all the possible dynamics when each gene is updated randomly, which is equivalent to calculating the transition graph. Furthermore, the logical formalism did not allow direct incorporation of diffusion and interaction of growth factors with extracellular matrix proteins. Previous models of signalling in the growth plate have explicitly modelled diffusion, whereas in the current work diffusion is modelled implicitly by changing the value of nodes representing diffusion [58]–[62].

In this work we have aimed to simulate the gene network that drives chondrocyte differentiation in the fetal growth plate. This model shows how genes interact to regulate and modify the differentiation state of a cell. Despite hiatuses in our current understanding of growth plate regulation the model provides a feasible representation of the governing regulatory apparatus. The rigid description of gene interactions allows testing the plausibility of hypotheses on gene interactions *in silico*. Hence a modelling approach helps in the formulation and subsequently rejection or corroboration of putative gene interactions in the context of an elaborate gene network, which in its intricacy is capable of defying human intuition.

The model introduced has further practical uses in the context of tissue engineering (TE). Currently, TE of bones uses a poorly characterized process in which the quantity and quality of the produced bone are low [63]. An alternative would be to use a biomimetic process growing bones via endochondral ossification, the natural route for long bone development. The developmental process is robust and behaves as an autoregulatory module. In accordance with the novel paradigm of “developmental engineering” these characteristics are highly desirable in an in vitro tissue engineering process [64]. The logical model can be instructive to design such a process, both in improving process robustness, observability and controllability and can hence assist in overcoming regulatory hurdles [65].

## Supporting Information

Material S1 Complete list of references for the different interactions in the logical model with indication of the (animal) model species used.
(PDF)

Material S2 Model files used to generate the results
(ZIP)

Material S3 Set-up and details of the ODE system used to assess stability of the logical model
(PDF)

Material S4 Results for conditional knock-outs in the Wnt and TGFb pathway and for the case where the perichondrium was removed.
(PDF)
