# Supplementary material for: Relating the Chondrocyte Gene Network to Growth Plate Morphology: From Genes to Phenotype
Source: PLoS One. 2012 Apr 30;7(4):e34729. doi: 10.1371/journal.pone.0034729 (PMC3340393; doi:10.1371/journal.pone.0034729)
Supplement: Material S1 — Complete list of references for the different interactions in the logical model with indication of the (animal) model species used. (PDF) [file pone.0034729.s001.pdf]

Active node	Activated node	Reference	System
Sox9	Sox9	[6;40]	Chick chondrocytes
bFGF	NF-κβ	[59]	Human chondrocytes
BMP	Ihh	[18;49;57;91;92]	Chicken/mice embryo (fetal growth plate)
BMP	Col-X	[1;91]	ATDC5 (mouse chondrogenic cell line)/ chicken chondrocytes (in vivo)
BMP	BMPR	[92]	Canonical
BMP	FGFR1	[92]	Mouse chondrocytes
BMP	STAT1	[92]	Mouse chondrocytes
BMP	GATA4	[58;65]	Mouse cardiomyocytes (P19CL6)
BMP2-4-7	Runx2	[72]	Human C2C12, (mouse premyoblasts), C3H10T1/2 (mouse mesenchymal)n human bone marrow derived cells, RoS 17,2.8 (rat osteosarcoma)
BMP6	Col-X, AP	[19;72]	Chick sternal cephalic chondrocytes
BMP pathway	R-smad	[1;18]	Canonical
BMPR	p38 kinase	[64;75;91]	Mouse embryonic fibroblasts (MEF)
cAMP	PKA	[49]	Canonical
CCND1	Runx2	[95]	Rat chondroprogenitor (RCJ3.1C5.18), C3H10T1/2
ERK 1/2	R-smad	[37;71]	Mouse lung, C2C12, MEFs
ERK1/2	Runx2	[9;28;89]	Human MSC, MC3T3 preosteoblasts (mouse)
FGF	Ras	[17]	Canonical
FGF	ERK	[48;91;92]	Chick embryo, mouse chondrocytes
bFGF	BMP7	[38;56]	Mouse limb bud
FGF18	FGFR3	[22;46;49;62]	Mice growth plate in vivo
FGF18	Ihh	[18;46]	Mice growth plate in vivo
FGFR1	NF-κβ	[73]	C2C12, T1/2, MC615 (chondrogenic mouse limb)
FGFR3	STAT1	[18;63;69]	Rat chondrosarcoma, murine primary chondrocytes
FGFs	Wnts	[18;80]	Mouse and chick limb buds
GATA4	MEF2C	[12]	Mouse anterior heart field in vivo
Gli	BMP2-7	[13]	HaCaT (Human adult

			low Calcium Temperature keratinocytes)
Gli	TGFβ1	[13]	“”
Gli	BMP6	[13]	“”
Gli	BMP2	[63;78]	Mouse and human BMP2 promoter
Gli2	Wnt11, Ptch1	[41;45]	HaCaT, MEFs
Gli2	MMP13	[13]	HaCaT
Gli2	PTHrP	[55;76]	Breast cancer, fetal mouse growth plate
Gli2	BMP4-7	[30]	Mouse and human promoter, osteoblastic Hos and renal COS-7 cells (human osteosarcoma and simian)
Gli2	FGFR1	[36]	Fetal growth plate
Gli2,3	Gli1	[41]	HaCaT (canonical?)
Gli3	PTHrP	[36;49]	Fetal growth plate
GSK3β	Gli	[27]	Drosophila, canonical
GSK3β	Gli2,3	[24]	Canonical
HDAC4	MEF2C	[35]	Mouse and chick limb bud cells
Ihh	PTHrP	[18;57;83]	Mouse fetal growth plate
Ihh	PPR	[50]	Mouse fetal growth plate (indirect)
Ihh	Ptch1, Gli1	[24]	canonical
Ihh	Wnt3a	[52]	Mouse limb explants, primary chondrocytes
Ihh pathway	Gli3	[1;18;49;51;88]	Canonical
MEF2C	Runx2	[5;49;54]	Mouse fetal growth plate
MEF2C	Col-X	[35;54]	Mouse and chick limb bud cells
MEF2C	Smaddlx5	[74;84]	Mouse branchial arch (enchancer element)
NF-κβ	Smad7	[7;11;53]	Mv1Lu, COS (simian), and NIH-3T3 (murine fibroblast cell lines)
NF-κβ	BMP2	[14;78;87]	MTC-23 (mouse chondrogenic cell line), mouse fetal growth plate
NF-κβ	Sox9	[34;73]	C2C12, C3H10T1/2, MC615
NF-κβ	MMP13	[59]	Human articular chondrocytes
Nkx3.2	Runx2	[18;49;67;90]	C3H10T1/2, murine (rib) chondrocytes, chick embryo explant
Noggin	BMP	[1;91]	canonical
p38 kinase	Sox9	[64]	MEFs
PKA	Sox9	[23;39;40]	Chicken primary chondrocytes, COS-7, RCS (rat

			chondrosarcoma)
PKA	Gli2,3	[24;85]	canonical
PKA	Col-X	[68]	Human and primary bovine chondrocytes
PKA	HDAC4	[35]	Mouse and chick limb bud cells
PKA	CCND1	[95]	Mouse fetal growth plate, COS, C3H10T1/2, RCJ3.1C5.18
PKA	Runx2	[43]	Chick sternal chondrocytes
PTHrP	cAMP	[23]	canonical
PTHrP	Nkx3.2	[49;67]	Chick embryo explant
R-smad	Dsh	[47]	C57BL/6 and ST2 mice primary bone marrow stromal cells
R-smad (BMP2)	PPR	[79]	C2C12 OB differentiation
Runx2	Col-X	[1;49]	Chick chondrocytes, functional sites in mice and chick promoter
Runx2	Ihh	[9;21;49;66;93]	Mouse fetal growth plate (+ promoter), MDA-MB-231 breast cancer cell line
FGFR1, FGF2-8	Runx2	[72;98]	MC3T3-E1 mouse calvarial cells,C3H10T1/2, in vivo
Runx2	FGF18	[28]	Mouse fetal growth plate
Runx2	MMP13	[21]	Mouse fetal growth plate
Runx2	MEF2C	[21]	Mouse fetal growth plate
Runx2-Smad complex		[9;26]	HeLa & mouse embryo
R-smad	Runx2	[61]	C2C12
Smad1	Col-X	[35]	Mouse and chick limb bud cells
Smad2,3	Col-II	[15]	Human MSCs and SW1353 (human chondrosarcoma)
Smad3	HDAC4	[28;29]	NIH3T3 (MEFs), ROS17/2.8, primary calvarial mouse osteoblasts
Smad3	Runx2	[3;11;72]	MC3T3-E1, ROS17/2.8, primary calvarial mouse osteoblasts
Smad3 β-catenin Lef/Tcf complex	Gli2	[10]	HaCaT, HepG2 (human)
Smad4	Smadcomplex	[1;18]	canonical
Smad7	R-smad	[1;18]	canonical
Smadcomplex	Runx2	[77;96]	C2C12
Smadcomplex	Smaddlx5	[33;44]	C2C12

Smaddlx5	Runx2	[44]	C2C12
Sox9	Col-II	[16;18]	Human MSCs, chondrocytes, SW1353 (canonical)
Sox9	Nkx3.2	[90]	C3H10T1/2, murine (fetal) chondrocytes,
Sox9	β-catenin	[20;81]	Mouse limb bud cells, murine primary chondrocytes , L cells (fibroblasts), CHO (chinese hamster ovary), HEK293 (Human embryonic kidney), COS1
Sox9	CCND1	[25;32]	Mouse fetal growth plate, SW1353 (human)
Sox9	Runx2	[97]	Mouse fetal growth plate, ROS17/2.8, COS7, human CMD1 cartilage (indirect)
STAT1	CKI	[92]	Mouse fetal growth plate
STAT1	Smad7	[11;82]	U4A cells (human fibrosarcoma), human monocytic leukaemia U937 cells, epidermoid carcinoma A431 cells
STAT1	Ihh	[70]	Mouse fetal growth plate
STAT1	PPR	[38;60]	Mouse fetal growth plate (indirect)
TGFβ	Sox9	[8;91]	Chick embryos
TGFβ	Ras	[11]	Canonical
TGFβ	Smad3	[2;28]	Canonical
TGFβ	CCND1	[4;42]	HCS-2/8, human chondrocyte-like cell line
TGFβ1	Smaddlx5	[44]	MC3T3-E1, ROS 17/2.8, and ST2 (mouse bone marrow osteogenic cells), ATDC5, C2C12 cells, C3H10T1/2, 3T3-L1 (adipogenic)
Wnt pathway	β-catenin	[49]	canonical
Wnt pathway	FGFs	[18;31]	Chick embryo
Wnt3a	Ras	[94]	Canonical (NIH3T3, L cells)
β-catenin	FGF8	[86]	Mouse *in vivo* (facial), chick limb bud

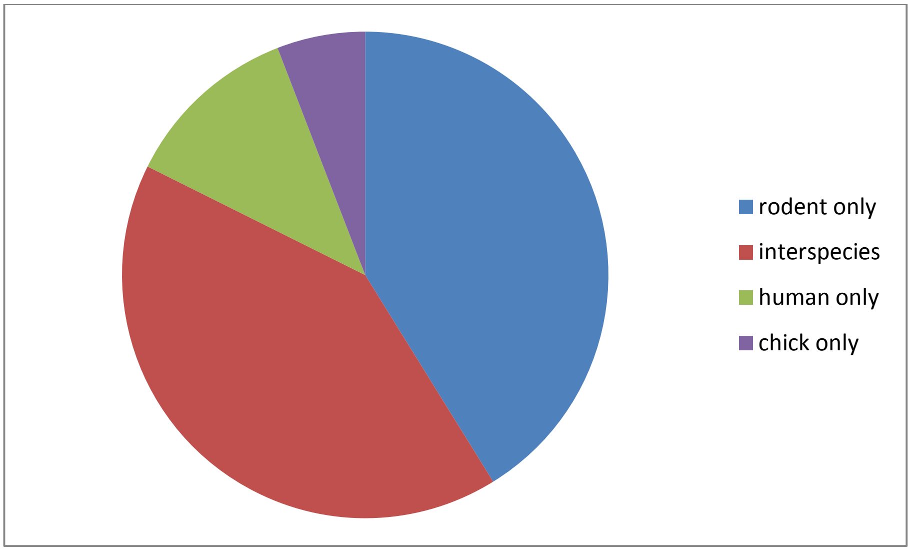

Figure 1: Overview of the sources of interactions by species. 82 % of the interactions were verified for mice.

Reference List

[1] S.L. Adams, A.J. Cohen, L. Lassová, Integration of signaling pathways regulating chondrocyte differentiation during endochondral bone formation. J.Cell.Physiol. 213 (2007) 635-641.

[2] H. Akiyama, Control of chondrogenesis by the transcription factor Sox9. Modern Rheumatology 18 (2008) 213-219.

[3] T. Alliston, L. Choy, P. Ducy, G. Karsenty, R. Derynck, TGF-β-induced repression of CBFA1 by Smad3 decreases cbfa1 and osteocalcin expression and inhibits osteoblast differentiation. EMBO J 20 (2001) 2254-2272.

[4] Y. Arai, T. Kubo, K. Kobayashi, K. Takeshita, K. Takahashi, T. Ikeda, J. Imanishi, M. Takigawa, Y. Hirasawa, Adenovirus vector-mediated gene transduction to chondrocytes: in vitro evaluation of therapeutic efficacy of transforming growth factor-beta 1 and heat shock protein 70 gene transduction. J Rheumatol. 24 (1997) 1787-1795.

[5] M.A. Arnold, Y. Kim, M.P. Czubryt, D. Phan, J. McAnally, X. Qi, J.M. Shelton, J.A. Richardson, R. Bassel-Duby, E.N. Olson, MEF2C Transcription Factor Controls Chondrocyte Hypertrophy and Bone Development. Developmental Cell 12 (2007) 377-389.

[6] A. Augello, C. De Bari, The Regulation of Differentiation in Mesenchymal Stem Cells. Human Gene Therapy 21 (2010) 1226-1238.

[7] M. Bitzer, G. von Gersdorff, D. Liang, A. Dominguez-Rosales, A.A. Beg, M. Rojkind, E.P. B+Âttinger, A mechanism of suppression of TGFβ/SMAD signaling by NF-κβ/RelA. Genes & Development 14 (2000) 187-197.

[8] J. Chimal-Monroy, J. Rodriguez-Leon, J.A. Montero, Y. Gañan, D. Macias, R. Merino, J.M. Hurle, Analysis of the molecular cascade responsible for mesodermal limb chondrogenesis: sox genes and BMP signaling. Developmental Biology 257 (2003) 292-301.

[9] J. Cohen, Perspectives on RUNX genes: An update. American Journal of Medical Genetics Part A 149A (2009) 2629-2646.

[10] S. Dennler, J. André, F. Verrecchia, A. Mauviel, Cloning of the Human GLI2 Promoter. Journal of Biological Chemistry 284 (2009) 31523-31531.

[11] R. Derynck, Y.E. Zhang, Smad-dependent and Smad-independent pathways in TGF-β family signalling. Nature 425 (2003) 577-584.

[12] E. Dodou, M.P. Verzi, J.P. Anderson, S.M. Xu, B.L. Black, Mef2c is a direct transcriptional target of ISL1 and GATA factors in the anterior heart field during mouse embryonic development. Development 131 (2004) 3931-3942.

[13] T. Eichberger, V. Sander, H. Schnidar, G. Regl, M. Kasper, C. Schmid, S. Plamberger, A. Kaser, F. Aberger, A.M. Frischauf, Overlapping and distinct transcriptional regulator properties of the GLI1 and GLI2 oncogenes. Genomics 87 (2006) 616-632.

[14] J.Q. Feng, L. Xing, J.H. Zhang, M. Zhao, D. Horn, J. Chan, B.F. Boyce, S.E. Harris, G.R. Mundy, D. Chen, NFκβ Specifically Activates BMP-2 Gene Expression in Growth Plate Chondrocytes in Vivo and in a Chondrocyte Cell Line in Vitro. Journal of Biological Chemistry 278 (2003) 29130-29135.

[15] T. Furumatsu, M. Tsuda, N. Taniguchi, Y. Tajima, H. Asahara, Smad3 Induces Chondrogenesis through the Activation of SOX9 via CREB-binding Protein/p300 Recruitment. Journal of Biological Chemistry 280 (2005) 8343-8350.

[16] T. Furumatsu, M. Tsuda, K. Yoshida, N. Taniguchi, T. Ito, M. Hashimoto, T. Ito, H. Asahara, Sox9 and p300 Cooperatively Regulate Chromatin-mediated Transcription. Journal of Biological Chemistry 280 (2005) 35203-35208.

[17] A. Goldbeter, O. Pourquié, Modeling the segmentation clock as a network of coupled oscillations in the Notch, Wnt and FGF signaling pathways. Journal of Theoretical Biology 252 (2008) 574-585.

[18] M.B. Goldring, K. Tsuchimochi, K. Ijiri, The control of chondrogenesis. J.Cell.Biochem. 97 (2006) 33-44.

[19] C.D. Grimsrud, P.R. Romano, M. D'souza, J.E. Puzas, P.R. Reynolds, R.N. Rosier, R.J. O'Keefe, BMP-6 Is an Autocrine Stimulator of Chondrocyte Differentiation. J Bone Miner Res 14 (1999) 475-482.

[20] C. Hartmann, Transcriptional networks controlling skeletal development. Current Opinion in Genetics & Development 19 (2009) 437-443.

[21] J. Hecht, V. Seitz, M. Urban, F. Wagner, P.N. Robinson, A. Stiege, C. Dieterich, U. Kornak, U. Wilkening, N. Brieske, C. Zwingman, A. Kidess, S. Stricker, S. Mundlos, Detection of novel skeletogenesis target genes by comprehensive analysis of a Runx2-/- mouse model. Gene Expression Patterns 7 (2007) 102-112.

[22] W.A. Horton, C.R. Degnin, FGFs in endochondral skeletal development. Trends in Endocrinology & Metabolism 20 (2009) 341-348.

[23] W. Huang, X. Zhou, V. Lefebvre, C.B. de, Phosphorylation of SOX9 by cyclic AMP-dependent protein kinase A enhances SOX9's ability to transactivate a Col2a1 chondrocyte-specific enhancer. Mol.Cell Biol. 20 (2000) 4149-4158.

[24] J.M. Hyman, A.J. Firestone, V.M. Heine, Y. Zhao, C.A. Ocasio, K. Han, M. Sun, P.G. Rack, S. Sinha, J.J. Wu, D.E. Solow-Cordero, J. Jiang, D.H. Rowitch, J.K. Chen, Small-molecule inhibitors reveal multiple strategies for Hedgehog pathway blockade. Proceedings of the National Academy of Sciences 106 (2009) 14132-14137.

[25] T. Ito, N. Yadav, J. Lee, T. Furumatsu, S. Yamashita, K. Yoshida, N. Taniguchi, M. Hashimoto, M. Tsuchiya, T. Ozaki, M. Lotz, M. Bedford, H. Asahara, Arginine methyltransferase CARM1/PRMT4 regulates endochondral ossification. BMC Developmental Biology 9 (2009) 47.

[26] A. Javed, J.S. Bae, F. Afzal, S. Gutierrez, J. Pratap, S.K. Zaidi, Y. Lou, A.J. van Wijnen, J.L. Stein, G.S. Stein, J.B. Lian, Structural Coupling of Smad and Runx2 for Execution of the BMP2 Osteogenic Signal. Journal of Biological Chemistry 283 (2008) 8412-8422.

[27] J. Jia, K. Amanai, G. Wang, J. Tang, B. Wang, J. Jiang, Shaggy/GSK3 antagonizes Hedgehog signalling by regulating Cubitus interruptus. Nature 416 (2002) 548-552.

[28] J.H. Jonason, G. Xiao, M. Zhang, L. Xing, D. Chen, Post-translational Regulation of Runx2 in Bone and Cartilage. Journal of Dental Research 88 (2009) 693-703.

[29] J.S. Kang, T. Alliston, R. Delston, R. Derynck, Repression of Runx2 function by TGF-[beta] through recruitment of class II histone deacetylases by Smad3. EMBO J 24 (2005) 2543-2555.

[30] S. Kawai, T. Sugiura, Characterization of human bone morphogenetic protein (BMP)-4 and -7 gene promoters: activation of BMP promoters by Gli, a sonic hedgehog mediator. Bone 29 (2001) 54-61.

[31] Y. Kawakami, J. Capdevila, D. Büscher, T. Itoh, C.R. Esteban, J.C.I. Belmonte, WNT Signals Control FGF-Dependent Limb Initiation and AER Induction in the Chick Embryo. Cell 104 (2001) 891-900.

[32] Y. Kawakami, J. Rodriguez-León, J.C.I. Belmonte, The role of TGFβs and Sox9 during limb chondrogenesis. Current Opinion in Cell Biology 18 (2006) 723-729.

[33] Y.J. Kim, M.H. Lee, J.M. Wozney, J.Y. Cho, H.M. Ryoo, Bone Morphogenetic Protein-2-induced Alkaline Phosphatase Expression Is Stimulated by Dlx5 and Repressed by Msx2. Journal of Biological Chemistry 279 (2004) 50773-50780.

[34] T. Kobayashi, U.i. Chung, E. Schipani, M. Starbuck, G. Karsenty, T. Katagiri, D.L. Goad, B. Lanske, H.M. Kronenberg, PTHrP and Indian hedgehog control differentiation of growth plate chondrocytes at multiple steps. Development 129 (2002) 2977-2986.

[35] E. Kozhemyakina, T. Cohen, T.P. Yao, A.B. Lassar, Parathyroid hormone-related peptide represses chondrocyte hypertrophy through a protein phosphatase 2A/histone deacetylase 4/MEF2 pathway. Mol.Cell Biol. 29 (2009) 5751-5762.

[36] L. Koziel, M. Wuelling, S. Schneider, A. Vortkamp, Gli3 acts as a repressor downstream of Ihh in regulating two distinct steps of chondrocyte differentiation. Development 132 (2005) 5249-5260.

[37] M. Kretzschmar, J. Doody, J. Massagu, Opposing BMP and EGF signalling pathways converge on the TGF-[beta] family mediator Smad1. Nature 389 (1997) 618-622.

[38] H.M. Kronenberg, Developmental regulation of the growth plate. Nature 423 (2003) 332-336.

[39] H.M. Kronenberg, PTHrP and Skeletal Development. Annals of the New York Academy of Sciences 1068 (2006) 1-13.

[40] D. Kumar, A.B. Lassar, The Transcriptional Activity of Sox9 in Chondrocytes Is Regulated by RhoA Signaling and Actin Polymerization. Molecular and Cellular Biology 29 (2009) 4262-4273.

[41] S. Laner-Plamberger, A. Kaser, M. Paulischta, C. Hauser-Kronberger, T. Eichberger, A.M. Frischauf, Cooperation between GLI and JUN enhances transcription of JUN and selected GLI target genes. Oncogene 28 (2009) 1639-1651.

[42] T.F. Li, R.J. O'Keefe, D. Chen, TGF-beta signaling in chondrocytes. Front Biosci. 10:681-8. (2005) 681-688.

[43] T.F. Li, Y. Dong, A.M. Ionescu, R.N. Rosier, M.J. Zuscik, E.M. Schwarz, R.J. O'Keefe, H. Drissi, Parathyroid hormone-related peptide (PTHrP) inhibits Runx2 expression through the PKA signaling pathway. Experimental Cell Research 299 (2004) 128-136.

[44] Y.l. Li, Z.s. Xiao, Advances in Runx2 regulation and its isoforms. Medical Hypotheses 68 (2007) 169-175.

[45] R.J. Lipinski, J.J. Gipp, J. Zhang, J.D. Doles, W. Bushman, Unique and complimentary activities of the Gli transcription factors in Hedgehog signaling. Experimental Cell Research 312 (2006) 1925-1938.

[46] Z. Liu, J. Xu, J.S. Colvin, D.M. Ornitz, Coordination of chondrogenesis and osteogenesis by fibroblast growth factor 18. Genes & Development 16 (2002) 859-869.

[47] Z. Liu, Y. Tang, T. Qiu, X. Cao, T.L. Clemens, A Dishevelled-1/Smad1 Interaction Couples WNT and Bone Morphogenetic Protein Signaling Pathways in Uncommitted Bone Marrow Stromal Cells. Journal of Biological Chemistry 281 (2006) 17156-17163.

[48] J.S. Lunn, K.J. Fishwick, P.A. Halley, K.G. Storey, A spatial and temporal map of FGF/Erk1/2 activity and response repertoires in the early chick embryo. Developmental Biology 302 (2007) 536-552.

[49] E.J. Mackie, Y.A. Ahmed, L. Tatarczuch, K.S. Chen, M. Mirams, Endochondral ossification: How cartilage is converted into bone in the developing skeleton. The International Journal of Biochemistry & Cell Biology 40 (2008) 46-62.

[50] H.E. MacLean, H.M. Kronenberg, Localization of Indian hedgehog and PTH/PTHrP receptor expression in relation to chondrocyte proliferation during mouse bone development. Development, Growth & Differentiation 47 (2005) 59-63.

[51] Y. Maeda, E. Schipani, M.J. Densmore, B. Lanske, Partial rescue of postnatal growth plate abnormalities in Ihh mutants by expression of a constitutively active PTH/PTHrP receptor. Bone 46 (2010) 472-478.

[52] K.K. Mak, H.M. Kronenberg, P.T. Chuang, S. Mackem, Y. Yang, Indian hedgehog signals independently of PTHrP to promote chondrocyte hypertrophy. Development 135 (2008) 1947-1956.

[53] J. Massagué, J. Seoane, D. Wotton, Smad transcription factors. Genes & Development 19 (2005) 2783-2810.

[54] T.A. McKinsey, C.L. Zhang, E.N. Olson, MEF2: a calcium-dependent regulator of cell division, differentiation and death. Trends in Biochemical Sciences 27 (2002) 40-47.

[55] D. Miao, H. Liu, P. Plut, M. Niu, R. Huo, D. Goltzman, J.E. Henderson, Impaired endochondral bone development and osteopenia in Gli2-deficient mice. Experimental Cell Research 294 (2004) 210-222.

[56] E. Minina, C. Kreschel, M.C. Naski, D.M. Ornitz, A. Vortkamp, Interaction of FGF, Ihh/Pthlh, and BMP Signaling Integrates Chondrocyte Proliferation and Hypertrophic Differentiation. Developmental Cell 3 (2002) 439-449.

[57] E. Minina, H.M. Wenzel, C. Kreschel, S. Karp, W. Gaffield, A.P. McMahon, A. Vortkamp, BMP and Ihh/PTHrP signaling interact to coordinate chondrocyte proliferation and differentiation. Development 128 (2001) 4523-4534.

[58] K. Monzen, I. Shiojima, Y. Hiroi, S. Kudoh, T. Oka, E. Takimoto, D. Hayashi, T. Hosoda, A. Habara-Ohkubo, T. Nakaoka, T. Fujita, Y. Yazaki, I. Komuro, Bone morphogenetic proteins induce cardiomyocyte differentiation through the mitogen-activated protein kinase kinase kinase TAK1 and cardiac transcription factors Csx/Nkx-2.5 and GATA-4. Mol.Cell Biol. 19 (1999) 7096-7105.

[59] P. Muddasani, J.C. Norman, M. Ellman, A.J. van Wijnen, H.J. Im, Basic Fibroblast Growth Factor Activates the MAPK and NFκβ Pathways That Converge on Elk-1 to Control Production of Matrix Metalloproteinase-13 by Human Adult Articular Chondrocytes. Journal of Biological Chemistry 282 (2007) 31409-31421.

[60] M.C. Naski, J.S. Colvin, J.D. Coffin, D.M. Ornitz, Repression of hedgehog signaling and BMP4 expression in growth plate cartilage by fibroblast growth factor receptor 3. Development 125 (1998) 4977-4988.

[61] R. Nishimura, K. Hata, S.E. Harris, F. Ikeda, T. Yoneda, Core-binding factor α1 (Cbfa1) induces osteoblastic differentiation of C2C12 cells without interactions with Smad1 and Smad5. Bone 31 (2002) 303-312.

[62] N. Ohbayashi, M. Shibayama, Y. Kurotaki, M. Imanishi, T. Fujimori, N. Itoh, S. Takada, FGF18 is required for normal cell proliferation and differentiation during osteogenesis and chondrogenesis. Genes & Development 16 (2002) 870-879.

[63] D.M. Ornitz, P.J. Marie, FGF signaling pathways in endochondral and intramembranous bone development and human genetic disease. Genes & Development 16 (2002) 1446-1465.

[64] Q. Pan, Y. Yu, Q. Chen, C. Li, H. Wu, Y. Wan, J. Ma, F. Sun, Sox9, a key transcription factor of bone morphogenetic protein-2-induced chondrogenesis, is activated through BMP pathway and a CCAAT box in the proximal promoter. J.Cell.Physiol. 217 (2008) 228-241.

[65] T. Peterkin, A. Gibson, R. Patient, Redundancy and evolution of GATA factor requirements in development of the myocardium. Developmental Biology 311 (2007) 623-635.

[66] J. Pratap, J.J. Wixted, T. Gaur, S.K. Zaidi, J. Dobson, K.D. Gokul, S. Hussain, A.J. van Wijnen, J.L. Stein, G.S. Stein, J.B. Lian, Runx2 Transcriptional Activation of Indian Hedgehog and a Downstream Bone Metastatic Pathway in Breast Cancer Cells. Cancer Research 68 (2008) 7795-7802.

[67] S. Provot, H. Kempf, L.C. Murtaugh, U.i. Chung, D.W. Kim, J. Chyung, H.M. Kronenberg, A.B. Lassar, Nkx3.2/Bapx1 acts as a negative regulator of chondrocyte maturation. Development 133 (2006) 651-662.

[68] S. Riemer, S. Gebhard, F. Beier, E. Pöschl, K. von der Mark, Role of c-fos in the regulation of type X collagen gene expression by PTH and PTHrP: Localization of a PTH/PTHrP-responsive region in the human COL10A1 enhancer. J.Cell.Biochem. 86 (2002) 688-699.

[69] M. Sahni, D.C. Ambrosetti, A. Mansukhani, R. Gertner, D. Levy, C. Basilico, FGF signaling inhibits chondrocyte proliferation and regulates bone development through the STAT-1 pathway. Genes & Development 13 (1999) 1361-1366.

[70] M. Sahni, R. Raz, J.D. Coffin, D. Levy, C. Basilico, STAT1 mediates the increased apoptosis and reduced chondrocyte proliferation in mice overexpressing FGF2. Development 128 (2001) 2119-2129.

[71] G. Sapkota, C. Alarcón, F.M. Spagnoli, A.H. Brivanlou, J. Massagué, Balancing BMP Signaling through Integrated Inputs into the Smad1 Linker. Molecular Cell 25 (2007) 441-454.

[72] L. Shum, G. Nuckolls, The life cycle of chondrocytes in the developing skeleton. Arthritis Res 4 (2002) 94-106.

[73] R. Sitcheran, P.C. Cogswell, A.S. Baldwin, NFκβ mediates inhibition of mesenchymal cell differentiation through a posttranscriptional gene silencing mechanism. Genes & Development 17 (2003) 2368-2373.

[74] L.A. Solomon, N.G. Bérubé, F. Beier, Transcriptional regulators of chondrocyte hypertrophy. Birth Defects Research Part C: Embryo Today: Reviews 84 (2008) 123-130.

[75] L.A. Stanton, T.M. Underhill, F. Beier, MAP kinases in chondrocyte differentiation. Developmental Biology 263 (2003) 165-175.

[76] J.A. Sterling, B.O. Oyajobi, B. Grubbs, S.S. Padalecki, S.A. Munoz, A. Gupta, B. Story, M. Zhao, G.R. Mundy, The Hedgehog Signaling Molecule Gli2 Induces Parathyroid Hormone-Related Peptide Expression and Osteolysis in Metastatic Human Breast Cancer Cells. Cancer Research 66 (2006) 7548-7553.

[77] M. Stock, F. Otto, Control of RUNX2 isoform expression: The role of promoters and enhancers. J.Cell.Biochem. 95 (2005) 506-517.

[78] T. Sugiura, Cloning and functional characterization of the 5'-flanking region of the human bone morphogenetic protein-2 gene. Biochem.J. 338 (1999) 433-440.

[79] A.R.G. Susperregui, F. Viñals, P.W.M. Ho, M.T. Gillespie, T.J. Martin, F. Ventura, BMP-2 regulation of PTHrP and osteoclastogenic factors during osteoblast differentiation of C2C12 cells. J.Cell.Physiol. 216 (2008) 144-152.

[80] D. ten Berge, S.A. Brugmann, J.A. Helms, R. Nusse, Wnt and FGF signals interact to coordinate growth with cell fate specification during limb development. Development 135 (2008) 3247-3257.

[81] L. Topol, W. Chen, H. Song, T.F. Day, Y. Yang, Sox9 Inhibits Wnt Signaling by Promoting β-Catenin Phosphorylation in the Nucleus. Journal of Biological Chemistry 284 (2009) 3323-3333.

[82] L. Ulloa, J. Doody, J. Massagué, Inhibition of transforming growth factor-[beta]/SMAD signalling by the interferon-[gamma]/STAT pathway. Nature 397 (1999) 710-713.

[83] C.C. van Donkelaar, R. Huiskes, The PTHrP-Ihh feedback loop in the embryonic growth plate allows PTHrP to control hypertrophy and Ihh to regulate proliferation. Biomech.Model.Mechanobiol. 6 (2007) 55-62.

[84] M.P. Verzi, P. Agarwal, C. Brown, D.J. McCulley, J.J. Schwarz, B.L. Black, The Transcription Factor MEF2C Is Required for Craniofacial Development. Developmental Cell 12 (2007) 645-652.

[85] B. Wang, J.F. Fallon, P.A. Beachy, Hedgehog-Regulated Processing of Gli3 Produces an Anterior/Posterior Repressor Gradient in the Developing Vertebrate Limb. Cell 100 (2000) 423-434.

[86] Y. Wang, L. Song, C.J. Zhou, The canonical Wnt/β-catenin signaling pathway regulates Fgf signaling for early facial development. Developmental Biology 349 (2011) 250-260.

[87] S. Wu, J.K. Flint, G. Rezvani, F. De Luca, Nuclear Factorκβ p65 Facilitates Longitudinal Bone Growth by Inducing Growth Plate Chondrocyte Proliferation and Differentiation and by Preventing Apoptosis. Journal of Biological Chemistry 282 (2007) 33698-33706.

[88] M. Wuelling, A. Vortkamp, Transcriptional networks controlling chondrocyte proliferation and differentiation during endochondral ossification. Pediatric Nephrology 25 (2010) 625-631.

[89] G. Xiao, D. Jiang, R. Gopalakrishnan, R.T. Franceschi, Fibroblast Growth Factor 2 Induction of the Osteocalcin Gene Requires MAPK Activity and Phosphorylation of the Osteoblast Transcription Factor, Cbfa1/Runx2. Journal of Biological Chemistry 277 (2002) 36181-36187.

[90] S. Yamashita, M. Andoh, H. Ueno-Kudoh, T. Sato, S. Miyaki, H. Asahara, Sox9 directly promotes Bapx1 gene expression to repress Runx2 in chondrocytes. Experimental Cell Research 315 (2009) 2231-2240.

[91] B.S. Yoon, K.M. Lyons, Multiple functions of BMPs in chondrogenesis. J.Cell.Biochem. 93 (2004) 93-103.

[92] B.S. Yoon, R. Pogue, D.A. Ovchinnikov, I. Yoshii, Y. Mishina, R.R. Behringer, K.M. Lyons, BMPs regulate multiple aspects of growth-plate chondrogenesis through opposing actions on FGF pathways. Development 133 (2006) 4667-4678.

[93] C.A. Yoshida, H. Yamamoto, T. Fujita, T. Furuichi, K. Ito, K.i. Inoue, K. Yamana, A. Zanma, K. Takada, Y. Ito, T. Komori, Runx2 and Runx3 are essential for chondrocyte maturation, and Runx2 regulates limb growth through induction of Indian hedgehog. Genes & Development 18 (2004) 952-963.

[94] M.S. Yun, S.E. Kim, S.H. Jeon, J.S. Lee, K.Y. Choi, Both ERK and Wnt/β-catenin pathways are involved in Wnt3a-induced proliferation. Journal of Cell Science 118 (2005) 313-322.

[95] M. Zhang, R. Xie, W. Hou, B. Wang, R. Shen, X. Wang, Q. Wang, T. Zhu, J.H. Jonason, D. Chen, PTHrP prevents chondrocyte premature hypertrophy by inducing cyclin-D1-dependent Runx2 and Runx3 phosphorylation, ubiquitylation and proteasomal degradation. Journal of Cell Science 122 (2009) 1382-1389.

[96] Y.W. Zhang, N. Yasui, K. Ito, G. Huang, M. Fujii, J.i. Hanai, H. Nogami, T. Ochi, K. Miyazono, Y. Ito, A RUNX2/PEBP2αA/CBFA1 mutation displaying impaired transactivation and Smad interaction in cleidocranial dysplasia. Proceedings of the National Academy of Sciences 97 (2000) 10549-10554.

[97] G. Zhou, Q. Zheng, F. Engin, E. Munivez, Y. Chen, E. Sebald, D. Krakow, B. Lee, Dominance of SOX9 function over RUNX2 during skeletogenesis. Proceedings of the National Academy of Sciences 103 (2006) 19004-19009.

[98] Y.X. Zhou, X. Xu, L. Chen, C. Li, S.G. Brodie, C.X. Deng, A Pro250Arg substitution in mouse Fgfr1 causes increased expression of Cbfa1 and premature fusion of calvarial sutures. Human Molecular Genetics 9 (2000) 2001-2008.
