# Supplementary material for: Relating the Chondrocyte Gene Network to Growth Plate Morphology: From Genes to Phenotype
Source: PLoS One. 2012 Apr 30;7(4):e34729. doi: 10.1371/journal.pone.0034729 (PMC3340393; doi:10.1371/journal.pone.0034729)
Supplement: Material S3 — Set-up and details of the ODE system used to assess stability of the logical model (PDF) [file pone.0034729.s003.pdf]

Addendum on ODE system to assess stability

This addendum details the system of ordinary differential equations we have used to assess the stability of the stable states found by the logical network.

It is possible that these singleton stable states would not be stable in a continuous analogue of the system. An analytical procedure to detect stability of these singletons is available [8], however given the relatively high number of nodes and thresholds in our model we opted for a numerical approach.

We adapted the continuous framework for Boolean networks to one more suited for multivalue logic. We could not employ a single conversion method, but equalized the behaviour of the continuous function and that of its logical counterpart, i.e. some functions have dominant inhibitors whereas others use a weighted sum approach (so one inhibitory function isn't dominating two activating ones).

The value of all nodes is normalized to allow for a direct comparison with the results of the logical framework. In these equations below, we have used a threshold of ½ to discriminate between the 0 and 1 levels of activation. For nodes with 3 levels (i.e. 0, 1 and 2), we have used ¼ and ¾ as thresholds. We assumed a constant relative degradation rate.

This gives rise to the following system of equations:

List of variables:

Wnt3a	Dsh	BmpR	R-smad	Ihh	Gli2	Bcat	LefTcf	Runx2	Sox9	PTHrP	PPR
1	2	3	4	5	6	7	8	9	10	11	12
**Col X**	**PKA**	**MEF2C**	**FGF**	**FGFR3**	**STAT1**	**Smad complex**	**Col II**	**Nkx3.2**	**ERK1/2**	**Ext PTHrP**	**TGFb**
**13**	14	15	16	17	18	19	20	21	22	23	24
**MMP13**	**Smad7**	**Smad3**	**extIhh**	**FGFR1**	**Smad3 Lef**	**NFkb**	**HDAC4**	**CCND1**	**Smad Dlx5**	**BMP**	
**25**	26	27	28	29	30	31	32	33	34	35	

List of equations:

$$\frac{dz_1}{dt} = S\left(z_5, q, \frac{1}{4}\right) - z_1$$

$$\frac{dz_2}{dt} = D\left(z_1 - \frac{S\left(z_4, q, \frac{1}{4}\right)}{2}, q\right) - z_2$$

$$\frac{dz_3}{dt} = S\left(z_{35}, q, \frac{1}{4}\right) - z_3$$

$$\frac{dz_4}{dt} = D\left(z_3 - \frac{S\left(z_{26}, q, \frac{1}{4}\right)}{2}, q\right) - z_4$$

$$\frac{dz_5}{dt} = D\left(\frac{2*z_9 + S\left(z_{19}, q, \frac{1}{4}\right) - 1}{2} + \frac{z_{28} - S\left(z_9, q, \frac{3}{4}\right)}{2}, q\right) - z_5$$

$$\frac{dz_6}{dt} = S\left(2*z_5 + z_{30}*\left(1 - S\left(z_7, q, \frac{3}{4}\right)\right), q, \frac{1}{2}\right) - z_6$$

$$\frac{dz_7}{dt} = D\left(z_2 - \frac{S\left(z_{10}, q, \frac{3}{4}\right)}{2}, q\right) - z_7$$

$$\frac{dz_8}{dt} = D(z_7, q) - z_8$$

$$\frac{dz_9}{dt} = D\left(\frac{2*z_8 + z_{34} + z_{22} + z_{15} - 2*z_{21} - z_{27} - z_{33} + \left(S\left(z_{22} + z_{34}, q, \frac{1}{2}\right) - 1\right) + \left(S\left(z_{15}, q, \frac{1}{2}\right) - 1\right)}{2}, q\right) - z_9$$

$$\frac{dz_{10}}{dt} = D\left(\frac{2*z_{14} + 2*z_{10} - 2*z_9 - z_{31} + S\left(z_{24} + z_3 - 1, q, \frac{1}{2}\right)}{2}, q\right) - z_{10}$$

$$\frac{dz_{11}}{dt} = D\left(\frac{S\left(z_{30} + z_6, q, \frac{1}{2}\right) - S\left(z_3, q, \frac{1}{4}\right) + z_{23}}{2}, q\right) - z_{11}$$

$$\frac{dz_{12}}{dt} = S\left(S\left(z_4, q, \frac{1}{4}\right) + S\left(S\left(z_9, q, \frac{1}{4}\right) + z_6, q, \frac{1}{2}\right) - 1 - z_{18}, q, \frac{1}{2}\right) - z_{12}$$

$$\frac{dz_{13}}{dt} = D\left(\frac{z_{15} + S\left(z_4, q, \frac{1}{4}\right) + 2*z_9 - 2 - z_{14}}{2}, q\right) - z_{13}$$

$$\frac{dz_{14}}{dt} = S\left(S\left(z_{11}, q, \frac{1}{4}\right) + z_{12} - 1, q, \frac{1}{2}\right) - z_{14}$$

$$\frac{dz_{15}}{dt} = S\left(z_9 + z_{19} - 2*z_{32}, q, \frac{1}{2}\right) - z_{15}$$

$$\frac{dz_{16}}{dt} = D\left(\frac{S\left(2*z_9 + 2*z_7, q, \frac{1}{2}\right)}{2} + S\left(z_{16}, q, \frac{3}{4}\right), q\right) - z_{16}$$

$$\frac{dz_{17}}{dt} = D\left(z_{16} - \left(1 - S\left(z_{10}, q, \frac{1}{4}\right)\right), q\right) - z_{17}$$

$$\frac{dz_{18}}{dt} = S\left(2*z_{29} + 2*z_{17} - S\left(z_3, q, \frac{3}{4}\right)*\left(1 - S\left(z_{29}, q, \frac{1}{4}\right)\right), q, \frac{1}{2}\right) - z_{18}$$

$$\frac{dz_{19}}{dt} = D\left(z_4 - \frac{z_{22}}{2}, q\right) - z_{19}$$

$$\frac{dz_{20}}{dt} = D\left(\frac{2 * z_{10} + z_{27} - 1}{2}, q\right) - z_{20}$$

$$\frac{dz_{21}}{dt} = \frac{S\left(2 * z_{10} + 2 * z_{11} * S\left(z_{12}, q, \frac{1}{2}\right)\right) + S\left(z_{11} * S\left(z_{12}, q, \frac{1}{2}\right), q, \frac{3}{4}\right)}{2} - z_{21}$$

$$\frac{dz_{22}}{dt} = S\left(2 * z_{29} + S\left(z_{17}, q, \frac{3}{4}\right), q, \frac{1}{2}\right) - z_{22}$$

$$\frac{dz_{23}}{dt} = 0$$

$$\frac{dz_{24}}{dt} = S\left(z_6, q, \frac{1}{2}\right) - z_{24}$$

$$\frac{dz_{25}}{dt} = S\left(2 * z_9 + z_{31} - z_6, q, \frac{1}{2}\right) - z_{25}$$

$$\frac{dz_{26}}{dt} = D\left(\frac{z_{31} + z_{18}}{2}, q\right) - z_{26}$$

$$\frac{dz_{28}}{dt} = 0$$

$$\frac{dz_{29}}{dt} = D\left(z_{16} - \left(1 - S\left(z_9, q, \frac{1}{4}\right)\right) - \frac{S\left(z_{19}, q, \frac{3}{4}\right)}{2}, q\right) - z_{29}$$

$$\frac{dz_{30}}{dt} = S\left(z_{27} + S\left(z_8, q, \frac{1}{4}\right) - 1, q, \frac{1}{2}\right) - z_{30}$$

$$\frac{dz_{31}}{dt} = S\left(z_{29}, q, \frac{1}{4}\right) - z_{31}$$

$$\frac{dz_{32}}{dt} = S\left(z_{27} + z_{14} - 1, q, \frac{1}{2}\right) - z_{32}$$

$$\frac{dz_{33}}{dt} = S\left(S\left(z_{10}, q, \frac{1}{4}\right) + z_{14} + z_6 - 2 * z_{18}, q, \frac{1}{2}\right) - z_{33}$$

$$\frac{dz_{34}}{dt} = S\left(2 * z_{19} - z_{27} - 2 * \left(1 - S\left(z_{15}, q, \frac{1}{2}\right)\right), q, \frac{1}{2}\right) - z_{34}$$

$$\frac{dz_{35}}{dt} = D\left(\frac{z_6 + z_{31}}{2}, q\right) - z_{35}$$

Where

$$D(z, q) = \frac{S\left(z, q, \frac{1}{4}\right) + S\left(z, q, \frac{3}{4}\right)}{2}$$

with $\mathrm{S(z, q, th)}$ a sigmoidal function with steepness q and threshold value th.

We used

$$S(z,q,th) = \frac{z^q(1+th^q)}{th^q+z^q} * Heaviside(z)$$

Which is a slightly modified version of the Hill function where the term $(1+\mathrm{th}^\mathrm{q})$ ensures that the value at z = 1 is always 1 and the Heaviside function assures that negative values are zero. An alternative is to use a logistic function, which gave qualitatively similar results.

This framework allowed us to relax the assumptions of the logical approach and to replace the Heaviside step functions with more gentle sigmoids. For the stable states shown in figure 4 of the manuscript, the stable state is not affected as long as the sigmoids are reasonably steep. Hence we can conclude that the results presented in our work are valid as long as the Boolean assumptions are not overly relaxed.

The table shows the Euclidean distance between the continuous stable state and its logical counterpart (this state was also normalized: [0,1,2] becomes [0,0.5,1] and [0,1] remains unaltered). Small distances indicate good correspondence between the logical and continuum approach.

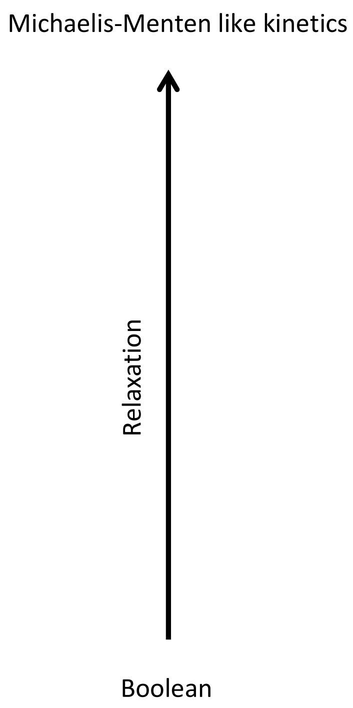

q	Resting	Proliferating	Hypertrophic
**1**	4,080	3,741	1,708
**2**	3,814	0,689	1,212
**3**	1,881	0,472	1,042
**4**	1,788	0,320	0,929
**5**	1,939	0,217	0,854
**6**	1,363	0,148	0,806
**7**	1,265	0,102	0,776
**8**	1,233	0,071	0,748
**9**	0,045	0,050	0,063
**10**	0,025	0,035	0,041
**11**	0,015	0,025	0,028
**12**	0,010	0,018	0,020
**13**	0,006	0,013	0,014
**14**	0,004	0,010	0,010
**15**	0,003	0,007	0,007
**16**	0,002	0,005	0,005
**17**	0,001	0,004	0,004
**18**	0,001	0,003	0,003
**19**	0,001	0,002	0,002
**20**	0,000	0,002	0,002

Clearly, these states are only stable up to a point. As the Boolean assumptions (step functions) are relaxed the discrepancy with the Boolean state slowly increases. This is to be expected since the system evolves to Michaelis-Menten kinetics as q approaches 1. By consequence, the way nodes influence each other will be fundamentally changed, as 2 thresholds will no longer be possible. We feel that these simulations show that the stable states are not an artefact of the Boolean framework and are present in analogous continuous model in a relevant region of the parameter space.
