# Supplementary material for: Relating the Chondrocyte Gene Network to Growth Plate Morphology: From Genes to Phenotype
Source: PLoS One. 2012 Apr 30;7(4):e34729. doi: 10.1371/journal.pone.0034729 (PMC3340393; doi:10.1371/journal.pone.0034729)
Supplement: Material S4 — Results for conditional knock-outs in the Wnt and TGFb pathway and for the case where the perichondrium was removed. (PDF) [file pone.0034729.s004.pdf]

Wnt mutants

Since complete deletion of β-catenin would result in the absence of osteoblast formation, the gene must be deleted in chondrocytes specifically [1]. Mice in which β-catenin has been deleted in chondrocytes are characterized by inhibited cell proliferation and faster maturation [2]. Conversely, chicken limbs where a stabilized form of β-catenin is expressed in chondrocytes showed shorter cartilage elements and ectopic FGF8 expression. The expression levels of other paracrine factors like Ihh and PTHrP were not influenced [3].

The results for both β-catenin mutant networks are given in table 1. For the activated β-catenin mutant, the resting zone situation is almost unaltered, since too many negative regulatory systems reduce Runx2 activity. Due to increased FGF expression the proliferative chondrocytes will not express CCND1 and consequently will proliferate slower. The Sox9 activity in this zone will also be lower than normal. Once the chondrocyte exits the PTHrP signalling range, hypertrophy will result. In this transition a reduced amount of Ihh secretion is predicted. This result is at odds with (semi-quantitative) measurements in mutant mice.

For the conditional β-catenin KO the resting zone is hard to represent. Since the alternative method to express PTHrP through Smad3 and Lef1 is not possible here due to the β-catenin KO, it is assumed that Gli2 is active here but does not induce BMP. Under this assumption the state in the resting zone is not markedly different from the wild type stable state. The proliferating zone is not different either, the Sox9-gene program still dominates here. When the chondrocytes exit PTHrP influence however, they will fail to undergo hypertrophy, although MEF2C is expressed. Hypertrophy will eventually ensue as more FGF ligands are

secreted by the adjacent perichondrium cells. Hence the β-catenin$^{-/-}$ mutant ultimately enters a Runx2-controlled program, but Runx2 is not as strongly expressed as in normal hypertrophy. This will probably result in a slower maturation, as observed in mutant mice. In the network hypertrophy is simplified to the expression of a limited number of currently known transcription factors that have been identified to be crucial to chondrocyte maturation. However, chondrocyte maturation is unlikely to be entirely encompassed by this limited set of transcription factors. Their expression or inhibition as a consequence of certain inputs might therefore better be interpreted as a speeding or slowing of chondrocyte differentiation rather than a total absence of hypertrophy. It should also be noted that though the resting zone states are not different in the model it can be expected that more Runx2 protein, though inactive, will be produced in the mutant with activated β-catenin.

TGFβ mutant

To test the influence of TGFβ transgenic mice with a dominant negative TβII receptor form were created. These mice exhibited an increase in collagen X expression in the growth plate. Furthermore, the cartilage in their joints had been replaced with bone or hypertrophic cartilage and the mice exhibited a phenotype similar to osteoarthritis [4]. This may be due to loss of TGFβ signalling in the mesenchyme [5]. Instead, the loss of TGFβ signalling might encourage chondrocytes to undergo terminal differentiation.

For the resting zone the same assumptions as in the β-catenin KO network have been made. In the proliferative stable state the collagen II secretion is diminished and MEF2C and Dlx5 are expressed ectopically which may indicate a leaning towards early hypertrophy. However, Sox9 expression is still dominant. The situation for the hypertrophic zone is

identical to the one in the wild type network. During the prehypertrophic phase Runx2 expression will be higher than normal at this point. However, the network does not predict higher Ihh activity (as seen in mutant mice [4]) as its node is already maximal. On the other hand, the output of collagen X is increased in this phase. The stable states are shown in table 2.

Perichondrium removal

The model detailed in this work describes the growth plate as an autoregulatory system. However, the origin of ligand's expression is not always clear as research of growth factors in the growth plate has focused more on their effects. The limited data available on BMP expression is testimony to this fact. A more common way to model gene regulatory networks is to only include transcription factors and receptors, leaving out ligand expression. This of course is not consistent with the view of the growth plate as an autonomous module. Nonetheless, many ligands appear to be primarily expressed in the perichondrium (e.g. FGF, certain Wnts, some BMPs [6]), supporting that the growth plate core is partly dependent on these signals.

With this adapted network without ligand regulation the removal of the perichondrium can be simulated. The perichondrium is vital in controlling chondrocyte maturation and proliferation [7]. Many of the growth factors regulating the growth plate are paracrine signals from the perichondrium. Therefore chondrocyte development will be altered when perichondrial signalling is lost, as is the case in an *in vitro* culture. Amongst the signals that will presumably be lost are TGFβ, some Wnt ligands and PTHrP [3,8,9]. Although Wnt signals are lost, Wnts expressed in the perichondrium include the noncanonical Wnt5a while

Wnt in chondrocytes, such as Wnt4, appears to be canonical. Hence Wnt signalling as modelled will still be present. Noncanonical Wnts normally slow chondrocyte maturation by interfering with β-catenin transcription of Runx2 [3].

If this is simulated in the model, the chondrocytes will undergo hypertrophy as no PTHrP or TGFβ is being produced. For the resting chondrocytes the network behaves just as in a TGFβ null situation. This result is consistent with the fact that Ihh normally acts on the perichondrium to initiate a negative feedback loop that regulates the hypertrophic differentiation process [8]. These results predict an inhibitory role of the perichondrium on the cartilage core, controlling hypertrophy. In light of this chondrocytes in an *in vitro* environment will undergo hypertrophy much faster compared to in the controlled *in vivo* environment. In an experiment with a chicken bone, an increased proliferation and differentiation of chondrocytes was seen after the perichondrium was removed. The researchers also noticed that the cartilage could no longer be divided into distinct zones as the expression of Ihh became diffuse [10]. The network furthermore has a higher expression of Ihh in these hypertrophic chondrocytes, since both the Smad complex and Runx2 are active. The stable state of growth plate chondrocytes without perichondrial influence can be viewed in table 3.

Table 1. Stable states of the β-catenin mutant networks.

	**Growth factors**						**Transcription factors**		
**β-catenin+**	**BMP**	**βcat**	**TGFβ**	**FGF**	**Ihh**	**PTHrP**	**Sox9**	**Runx2**	**Gli2**
**resting zone**	0	2	1	2	0	2(+ext)	1	0	0
**proliferative**	1	2	1	2	1(ext)	1(ext)	*1*	0	1
**hypertrophy**	2	2	1	2	1	0	0	2	1
**β-catenin-**	**BMP**	**βcat**	**TGFβ**	**FGF**	**Ihh**	**PTHrP**	**Sox9**	**Runx2**	**Gli2**
**resting zone**	0	0	1	0	1(ext)	2(+ext)	1	0	1
**proliferative**	1	0	1	0	1(ext)	1(ext)	2	0	1

**hypertrophy**	1	0	0	0	1(ext)	0	1	*0*	1
**external FGF**	2	0	0	*2*	1(ext)	0	0	*1*	1

Table 2. Stable states of the TGFβ mutant network.

The hypertrophic zone is unchanged.

	Growth factors						Transcription factors		
	**BMP**	**Wnt**	**TGFβ**	**FGF**	**Ihh**	**PTHrP**	**Sox9**	**Runx2**	**Gli2**
**resting zone**	0	1	0	0	1(ext)	2(+ext)	1	0	1
**proliferative**	1	1	0	1	1(ext)	1(ext)	2	0	1
prehypertrophic	1	1	0	1	2(+ext)	0	0	*2*	1

Table 3. The stable hypertrophic state of growth plate chondrocytes without perichondrium.

	Growth factors						Transcription factors		
	**BMP**	**Wnt**	**TGFβ**	**FGF**	**Ihh**	**PTHrP**	**Sox9**	**Runx2**	**Gli2**
**hypertrophy**	1	1	0	0	2	0	0	2	1

Reference List

1. Hill TP, Später D, Taketo MM, Birchmeier W, Hartmann C (2005) Canonical Wnt/β-Catenin Signaling Prevents Osteoblasts from Differentiating into Chondrocytes. Developmental Cell 8: 727-738. doi: 10.1016/j.devcel.2005.02.013.

2. Akiyama H, Lyons JP, Mori-Akiyama Y, Yang X, Zhang R, Zhang Z, Deng JM, Taketo MM, Nakamura T, Behringer RR, McCrea PD, de Crombrugghe B (2004) Interactions between Sox9 and β-catenin control chondrocyte differentiation. Genes & Development 18: 1072-1087.

3. Hartmann C, Tabin CJ (2000) Dual roles of Wnt signaling during chondrogenesis in the chicken limb. Development 127: 3141-3159.

4. Serra R, Johnson M, Filvaroff EH, LaBorde J, Sheehan DM, Derynck R, Moses HL (1997) Expression of a Truncated, Kinase-Defective TGF-β Type II Receptor in Mouse Skeletal Tissue Promotes Terminal Chondrocyte Differentiation and Osteoarthritis. The Journal of Cell Biology 139: 541-552.

5. Derynck R, Zhang YE (2003) Smad-dependent and Smad-independent pathways in TGF-[beta] family signalling. Nature 425: 577-584. 10.1038/nature02006.

6. Pogue R, Lyons K (2006) BMP Signaling in the Cartilage Growth Plate. In: Gerald PS, editors. Current Topics in Developmental Biology. Academic Press. pp. 1-48.

7. Colnot C, Lu C, Hu D, Helms JA (2004) Distinguishing the contributions of the perichondrium, cartilage, and vascular endothelium to skeletal development. Developmental Biology 269: 55-69. doi: 10.1016/j.ydbio.2004.01.011.

8. Vortkamp A, Lee K, Lanske B, Segre GV, Kronenberg HM, Tabin CJ (1996) Regulation of rate of cartilage differentiation by Indian hedgehog and PTH-related protein. Science 273: 613-622.

9. Alvarez J, Sohn P, Zeng X, Doetschman T, Robbins DJ, Serra R (2002) TGFβ2 mediates the effects of Hedgehog on hypertrophic differentiation and PTHrP expression. Development 129: 1913-1924.

10. Long F, Linsenmayer TF (1998) Regulation of growth region cartilage proliferation and differentiation by perichondrium. Development 125: 1067-1073.
